# Sensitivity of CNN image analysis to multifaceted measurements of neurite growth

**DOI:** 10.1186/s12859-023-05444-4

**Published:** 2023-08-24

**Authors:** Joseph T. Vecchi, Sean Mullan, Josue A. Lopez, Madeline Rhomberg, Annamarie Yamamoto, Annabelle Hallam, Amy Lee, Milan Sonka, Marlan R. Hansen

**Affiliations:** 1 Department of Molecular Physiology and Biophysics, Carver College of Medicine, University of Iowa, Iowa City, IA USA; 2 Iowa Institute for Biomedical Imaging, Electrical and Computer Engineering, University of Iowa, Iowa City, IA USA; 3 Department of Neuroscience, University of Texas-Austin, Austin, TX USA; 4 Department of Otolaryngology Head-Neck Surgery, Carver College of Medicine, University of Iowa, Iowa City, IA USA

**Keywords:** Neurite growth, Neuron morphology, Machine learning, Convolutional neural network, Explainable AI, Neurite guidance, High-content image analysis, Concept vector

## Abstract

Quantitative analysis of neurite growth and morphology is essential for understanding the determinants of neural development and regeneration, however, it is complicated by the labor-intensive process of measuring diverse parameters of neurite outgrowth. Consequently, automated approaches have been developed to study neurite morphology in a high-throughput and comprehensive manner. These approaches include computer-automated algorithms known as 'convolutional neural networks' (CNNs)—powerful models capable of learning complex tasks without the biases of hand-crafted models. Nevertheless, their complexity often relegates them to functioning as 'black boxes.' Therefore, research in the field of explainable AI is imperative to comprehend the relationship between CNN image analysis output and predefined morphological parameters of neurite growth in order to assess the applicability of these machine learning approaches. In this study, drawing inspiration from the field of automated feature selection, we investigate the correlation between quantified metrics of neurite morphology and the image analysis results from NeuriteNet—a CNN developed to analyze neurite growth. NeuriteNet accurately distinguishes images of neurite growth based on different treatment groups within two separate experimental systems. These systems differentiate between neurons cultured on different substrate conditions and neurons subjected to drug treatment inhibiting neurite outgrowth. By examining the model's function and patterns of activation underlying its classification decisions, we discover that NeuriteNet focuses on aspects of neuron morphology that represent quantifiable metrics distinguishing these groups. Additionally, it incorporates factors that are not encompassed by neuron morphology tracing analyses. NeuriteNet presents a novel tool ideally suited for screening morphological differences in heterogeneous neuron groups while also providing impetus for targeted follow-up studies.

**Supplementary Information:**

The online version contains supplementary material available at 10.1186/s12859-023-05444-4.

## Introduction

### Existing approaches to study neurite growth are inadequate

Understanding the mechanisms that govern neurite growth and branching, during development and in response to injury or disease, represents a fundamental objective for cellular and molecular neuroscience [1–3]. Neurite growth creates complex morphologies that are challenging to analyze holistically. The inherent heterogeneity of many neuronal populations further complicates this analysis [4, 5]. Though difficult, deciphering the mechanisms that regulate neurite growth is essential to understand neural development, treat or prevent peripheral neuropathies [6], direct neurite growth towards targets such as a neural electrode [7, 8], and understand pathophysiological underpinnings of neurological diseases [9]. Thus, it is essential to create and investigate methodologies that comprehensively analyze neurite growth and morphology.

Motivated by this need, we developed NeuriteNet, a machine learning image analysis program, that provides a rapid classification and scoring of neurite growth and morphology without the potential bias and limitations of manually formulated input parameters [10]. This tool was developed to study subtle, yet biologically significant, morphological changes resulting from experimental manipulations or genetic models that are difficult to detect by traditional methods of analysis and thus suggest features of neuron growth for further exploration [11]. Researchers may not detect these subtle differences because they (1) restrict the analyses to a subpopulation of neurons, (2) decrease the sample size to facilitate a low throughput, but more thorough method (i.e., Sholl analysis), or (3) use a higher throughput method that only assesses a narrow, predetermined metric (i.e., measuring longest axon). Furthermore, traditional approaches to manually measure neuron morphology are insufficient as they fail to simultaneously assess precise details and diverse indicators of differences in neuron morphology [12, 13]. For example, Sholl analysis presents a thorough picture of neurite growth and branching, however, this approach omits quantifying neurite alignment or the orientation of neurites relative to one another. An analysis tool that assesses both the precise micro-details of neurite morphology while also considering the macro-scale neuron orientation would be ideal.

### Convolutional Neural Networks represent a promising tool for analyzing neurite growth

Machine learning approaches fill this niche since they are high-throughput and thorough, while holistically analyzing neuron morphology without the need for manually crafted input parameters [14, 15]. This is exemplified by convolutional neural networks (CNNs) being successfully applied to similar image analysis tasks, including detecting or classifying cancer in tissue sections, CT scans, or mammograms [16–18]. Similar to neuron morphology, analysis of these structures in images also requires the simultaneous assessment of micro and macro features, while balancing throughput with thoroughness [19].

In such, similar CNNs have been applied to neuron morphology analysis [20, 21], including our work on NeuriteNet, a CNN image classification model that compares neuronal populations based on differences in neurite growth patterns [10]. Here, we build upon that work by providing further validation of CNN models via studying the sensitivity of NeuriteNet to specific and objective measurements of neurite growth morphology. Specifically, to further the application of CNNs in studying neuron growth morphology, our purpose here is to study the internal patterns of activation used by NeuriteNet to classify images and evaluate the relationship between the model's classification decision and relevant measurements of neuron morphology.

### CNNs need to be validated for this application

Despite their incredible representational power, CNNs function as “black boxes.” This means that it is hard for users to know the basis of a model’s classification or scoring of an image [22]. This lack of clarity is a major drawback for image analysis tasks where it is essential to know how or why the treatment groups differ from one another. To this end, new approaches are being developed that help explain the factors that drive CNN image classification decisions in the field of explainable AI and automated feature selection [23–25]. In our previous work, we used XRAI (eXplantation with Ranked Area Integrals) saliency maps to generate overlays to indicate what regions or aspects of a given image were important for dictating the model’s classification prediction of that image [10, 26]. This offered subjective insights into the components of neuronal morphology the model determined most distinguishing and provided validation of NeuriteNet in that the saliency map highlighted the expected distinctive features of each neuron group.

While saliency maps offer some qualitative explanation of CNN function, connecting NeuriteNet’s image classification and scoring to quantified neuron measurements would further corroborate and strengthen the use of CNNs for investigations into neurite growth morphology [27, 28]. However, while this kind of analysis can be relatively intuitive when performed on deep learning models trained using tabular data, where the input features are discrete values that can be directly examined [SHAP, LIME] [29, 30] It is much more difficult to do so for models trained using image data, where the input features can be millions of pixels that the model has learned to extract higher-order features from. Thus, in this study, we seek to further validate NeuriteNet by first using concept vectors [TCAV] to determine the relationship between these higher-order features and the morphological measurements generated from semi-automated, full-neurite tracing [31, 32]. Then, we use these concepts vectors to evaluate how the morphological differences described by these measurements effect NeuriteNet’s classification of the images. In our assessment of the relationship between these image analysis approaches, NeuriteNet demonstrates that its analysis is (1) more accurate in its classification of neurons by target categories than comparable methods and is capable of outputting a score related to its classification decision, (2) correlated with quantified the measurements of neuron morphology generated from neurite tracing data which differentiate the treatment groups, and (3) sensitive to variations in neuron tracing measurements when assessing the patterns of activation of the neural network model. Thus, in this work, we show that a CNN image analysis model focuses on and is activated by relevant features of neuron morphology in its classification as well as generates quantitative scoring that is sensitive to changes in neurite growth patterns.

## Methods

### Animals

All procedures involving animals were conducted in accordance with the NIH Guide for the Care and Use of Laboratory Animals and were approved by the University of Iowa Institutional Animal Care and Use Committee. All mice were maintained on a C57BL/6 (Envigo) background, housed in groups on a standard 12:12 h light: dark cycle with food and water provided ad libitum, and used at 4–6 weeks of age. Mice used were anesthetized with isoflurane prior to decapitation and desired tissue harvested. The data set used consisted of 3 experimental replicates for each condition studied across 3 independent variables (Male vs. Female, WT vs. CaBP1 KO (C-KO), and Unpatterned vs. Patterned). The characterization of C-KO mice (RRID: MGI: 5780462) has been described previously [33].

### Micropatterned substrates

Topographically micropatterned substrates consisting of repeating rows of ridges and grooves were generated using photopolymerization as previously described [34, 35]. In short, a monomer solution (40 wt% hexyl methacrylate (HMA, Aldrich) and 59 wt% 1,6-hexanediol dimethacrylate (HDDMA, Aldrich), 1 wt% of 2,2-dimethoxy-2-phenylacetophenone (DMPA, BASF)) was pipetted onto a silane coupled cover glass square and evenly dispersed by placing a glass-chrome Ronchi ruled photomask (Applied Image Inc.) on top, while unpatterned substrates were generated by placing a square glass slide on top instead. Samples were then exposed to 365 nm light at an intensity of 16mW/cm^2^ using a high-pressure mercury vapor arc lamp (Omnicure S1500, Lumen Dynamics, Ontario, Canada) to polymerize the monomer solution. This creates a topographically micropatterned substrate since the opaque portions of the mask selectively shade monomer from exposure to UV radiation thereby modulating the rate of the polymerization locally to generate features on the surface. This modulation creates raised features or ridges underneath transparent bands where UV light intensity and the polymerization rate are highest [36].

These patterned substrates direct the growth of various cells and neurons, including dorsal root ganglion root neurons (DRGNs). These neurons have an innate ability to sense and extend their neurites in directions corresponding to these biophysical features present in the patterned substrates [37]. Additionally, various geometries of topographical substrates can be generated by varying the photomask (to change periodicity of the ridges and troughs) or UV light exposure (to change feature amplitude) [35, 38]. A single micropattern geometry (50 µm periodicity and 4 µm amplitude) was chosen for this study since this was expected to elicit moderate guidance of replated DRGN (rDRGN) neurites, whereby it is clear that the neurites are following the topographical features, but the strength of guidance could be improved upon or inhibited [35, 37].

### Neuron cultures

Dissociated DRGN cultures were prepared, as previously described from the 4–6-week-old mice [10, 39]. First, a 24 well polystyrene plate was coated with poly-l-ornithine solution (Sigma-Aldrich) for 1 h at RT. After which the surface was washed with sterile Milli-Q^®^ three time prior to a laminin solution (20 μg/ml, Sigma-Aldrich) being added and incubated overnight at 4 °C. After warming the coated well plate for 1 h at 37 °C, the freshly dissected DRGNs were cultured on this plate for 72 h prior to being replated. The replating procedure has been described previously [40]. In short, after a 1 min incubation with TrypLE Express (Thermo Fisher) warm media was used to gently triturate the culture surface and lift the adhered neurons. The resulting replated neurons were then cultured on the micropatterned or unpatterned HDDMA/HMA substrates for 24 h. After 24 h on the HDDMA/HMA surfaces, the cultures were fixed and labeled for NF200 via immunofluorescence, as previously described [10].

### Image processing

Digital epifluorescence images were captured using MetaMorph software (Molecular Devices) on a Leica DMIRE2 microscope (Leica Microsystem) with a Leica DFC350FX digital camera. All images were captured as greyscale images with 1024 × 1360 pixel resolution and saved as unsigned 8-bit PNG images with pixel values between 0 and 255. The pre-processing for the images was done using three steps. First, to decrease the influence of extreme points of brightness, the image intensities were clipped so that any pixel values from 50 to 255 were set to only 50. Second, to remove any lighting-based background features, the magnitude of the image gradients was estimated using the combination of horizontal and vertical Sobel kernels, equation below [41].

$$G= \sqrt{G{x}^{2}+ G{y}^{2}}$$

Third, each image was rescaled to values between 0 and 1 based on image-level intensity ranges before and after the gradient magnitude estimates were performed.

### Semi-automated measurements of neuron morphology

For this study, rDRGNs were used due to their vigorous neurite growth and simpler morphology [40]. This morphology enabled large numbers of DRGNs to be traced via semi-automated approaches for this analysis. The first image set for this study consisted of 3 culture replicates of rDRGNs across 3 independent binary variable conditions: substrate (unpatterned or patterned), genotype (WT or C-KO), and sex.

In this study we focused our analyses on the substrate comparison. Thus 10 random neurons from each experimental replicate of each treatment group were selected for full neurite tracing analysis using NeuronJ [31], creating 240 total traced neurons for the dataset. From the tracing data, we generated data describing 6 aspects of neuron morphology, (1) Total Length (sum length of all traced branches), (2) Branch Length Variation (variance in branch lengths of a neuron), (3) Number of Branches (count of all branches traced), (4) Branching Density (number of branches per 100 µm of neurite length), (5) Alignment Index (total neurite length divided by total length in direction of pattern), and (6) Alignment Variation (variance in alignment index for each branch of a neuron).

The second image set consisted of 3 culture replicates from WT male mice of rDRGNs treated either before or after replating with Nocodazole (50 µM). In this image set, only the length of the longest neurite was measured, as described previously [39].

### Training NeuriteNet

We used the previously described NeuriteNet architecture for each of our experiments [10]. The network makes use of the source image resolution of 1024 × 1360 to best preserve any fine features of the neurons that would otherwise be lost during any down-sampling process. The network is comprised of two main structural features: a core of alternating strided and un-strided convolution layers and a series of side branches that connect different layers of spatial representation. Each pair of convolution layers that comprise the core of the model start with a convolution with a stride of 2 × 2, decreasing the spatial representation by half. The first pair of these layers uses 4 feature channels, and then each of the following 4 pairs of convolution layers doubles this number up to 64 feature channels.

After each pair of convolution layers, a side-branch of the network is created. These branches use a max-pooling operation followed by an un-strided convolution to pass forward each spatial level of feature representation. The size of each pool is relative to the point of the core that it branched from so that each branch results in a feature map of the same spatial dimensions with 16 feature channels. The final layer of the core of the model and the 4 side-branches of the model are concatenated together to form a single feature map with 32 × 43 spatial dimensions and 128 feature channels. This is passed through one final convolution layer to further merge the feature maps. A global average pooling layer is applied so that each of the 128 feature channels are reduced to a single representative value, resulting in a 128-feature vector. The pooling layer is followed by a fully-connected layer with 128 nodes. Finally, a 20% dropout layer followed by a final fully-connected layer with a sigmoid activation is used to generate the final classification decisions. For each classification decision, NeuriteNet outputs a continuous score with a possible range from 0 to 1. Each output decision is a classification between two classes, so scores approaching 0 indicate a stronger decision towards the 0 class (Unpatterned and After) while scores approaching 1 indicate a stronger decision towards the class associated with 1 (Patterned and Before). As the model does not have prior knowledge about the training data, the decision to assign a class to 0 or 1 is arbitrary so long as the two classes are mutually exclusive. Additionally, the number of output nodes was dependent on the number of classification decisions being made in each experiment. This was either a single node for the experimental-condition classification predictions (Before vs After) or three nodes for the substrate, genotype, and sex comparison. Importantly, while we focused on the substrate comparison for this work, the inclusion of genotype and sex classification comparisons were used to provide NeuriteNet with all the information of the dataset and improve model performance. Requiring the model to learn information related to the two additional classification tasks forced it to encode a wider variety of features than if it were trained only for the substrate comparisons. Additionally, introducing the additional tasks as part of the training process acts to prevent the model from over-fitting on any single task and allows it to converge to a more generalizable set of weights.

To train our model, we divided our images into five cross-validation groups. For each fold of our validation, four of these groups were used to train our model and the left-out group was used to test the model. The images were separated into groups based on their experimental group so that no experimental group was present in both the training and test data for any of our models.

Each model was trained using a batch size of 4 images for 300 epochs, where one epoch presented each image to the model exactly once. We used binary cross-entropy as the loss function for our model and updated the weights using the Adam optimization algorithm [42] and a decaying learning rate. Our learning rate started at a value of 0.001 and was decreased by 10% every time the model had gone 5 epochs without a decrease in classification error. To prevent model over-confidence and over-fitting, scores were smoothed by 0.05 during training so that the “correct” values used to calculate classification error were 0.05 and 0.95 rather than 0 and 1.

As mentioned earlier, NeuriteNet does not simply classify an image, but rather assign a score to each image. This score gives the strength of the model’s classification decision for the given input image and represents how characteristic of a treatment it is based on the features learned by the model. For the classification based on substrate (Unpatterned vs. Pattern), this score will be referred to as Predicted Pattern Score with values closer to 0 indicating that the model more strongly associates the given image with the Unpatterned condition while values closer to 1 indicate a stronger association with the Pattern condition. Similarly, in the second classification comparison (Before vs. After), this score will be referred to as Predicted Treatment Score with values towards 0 indicating After and values towards 1 indicating Before.

### Training generic CNN classification model

We employ a “generic CNN” as a comparison for NeuriteNet, which we train and test using the same approach as NeuriteNet. The generic CNN has an identical architecture as NeuriteNet but lacks side-branches. The remaining structure of alternative strided and non-strided convolutions is a standard architecture that has been applied to many basic classification tasks. Using this generic CNN as a comparison allows for the assessment of the side branches which enable our model to simultaneously use features from multiple levels of spatial resolution.

### Classifying images by tracing data

As a comparison for the classification accuracy of the CNN approaches, we employed a machine learning approach to classify images based on their tracing data using Scikit-learn [43]. We classified the 240 neurons with tracing data via the following approach. First, tracing data were scaled in a standardized manner by using the QuantileTransformer function followed by the MinMaxScaler function. This approach was done to minimize the effect of outliers, preserve the unique distribution of each metric, and put all data in the same 0 to 1 scale. Then, as with NeuriteNet, data were split into 5 cross-validation groups and a standard RidgeClassifier model was trained on 4 folds and the omitted group was used to test the model. This was repeated 5 times to generate a classification result for all the images. A confusion matrix was generated to compare the three classification methods and ascertain the Kappa Statistic for each classification test [44].

### Saliency map overlays

To aid the interpretation of our trained networks, we generated saliency maps for our models’ predictions (Additional file 1: Fig. S1). Saliency maps determine the relative importance that each pixel of an input image has for the resulting prediction from a given deep learning network. We generated the saliency maps for our model using an adapted version of the XRAI attribution method [26]. The original XRAI implementation is a form of segment-based post-processing applied to a gradient-based method of saliency map acquisition [45]. Gradient-based saliency maps have been shown to often be overly sensitive to edges in natural images [46], and this issue is further exacerbated by the extraordinarily strong edges in our images. We instead opted to use an occlusion-based method to acquire the initial saliency maps which does not suffer the same limitations [47].

To generate a saliency map using occlusion, we first used our model to get a baseline predicted classification score for a given image. Next, we used a black box of varying sizes to occlude regions of our image. Finally, we used our model to get a predicted classification score for the occluded image. The difference between the predicted score of the occluded images and the baseline score was assigned to the occluded region of the original image to represent the approximate influence that the features in that region had on the baseline prediction. We repeated this occlusion iteratively across the image until the influence for all regions of the image had been determined. We used square black boxes with edge lengths of 20, 25, 30, and 35 pixels to examine different scales of feature representation, and each occlusion was performed so that there was a 50% overlap with neighboring regions. The final saliency map was determined by taking the average of all occlusion results for each region of the image.

While saliency maps allow for insight into how the model is influenced by different regions of the image, they are at a much lower resolution than the input images. Using gradient-based saliency maps for our model would have resulted in maps with a resolution of 32 × 43 pixels, and even the finest level of detail for our occlusion-based method still utilizes regions of 20 × 20 pixels for each step. To better associate the coarse-grained saliency maps with the fine details of the input images, we utilized the XRAI post-processing method. For the this approach, we used the Felzenswalb graph-based segmentation method to separate the image into many overlapping regions [48]. Since the segment boundaries often align with edges, such as those formed by the neurites, we dilated the generated segments by 5 pixels to better include the finer neurites. We then iteratively added each segment to a final attribution map ordered by the average gain in attribution for the region of the occlusion-based saliency map covered by the given segment. After each segment was added to the final map, any area it shared was removed from neighboring segments. This process was repeated until there were no segments remaining that covered at least a minimum of 400 pixels. Any regions of the final saliency map that were not covered by an added segment from the XRAI process were covered by the same region of the occlusion-only saliency map (Additional file 1: Fig. S1).

Two things that should be noted when interpreting saliency maps are that (1) saliency maps represent how the input image regions influence the model’s decision and (2) saliency maps show the relative influence of regions of an input image on the model’s decision. This first point means that physical components of the neurite morphology may not be represented in the saliency map if they were not influential in the model’s decision. Similarly, empty space in the image may be represented in the saliency map if it is an important feature for the model’s decisions. Empty space near a cell body may indicate a lack of branching and thus may be an important part of the model’s decision. The second point also means that saliency maps do not have a consistent scale across samples. Meaning to enable side-by-side comparison, each saliency map needs to be rescaled to a range from 0 to 1 before the map is presented for visual examination. This is done by dividing by the greatest absolute value present in the given map. While this is necessary to enable comparison, a single influential feature may end up suppressing other features in the saliency map, or conversely a lack of influential features may end up exaggerating the impact of other features. Saliency maps are powerful tools for evaluating relative importance of features and trends in how a CNN uses images to make decisions, but determining an absolute importance score for features in the input image is still an open question. Thus, for their optimal interpretation they should be complemented with other methods assessing CNN image analysis performance as well as their suggestions taken into account alongside the known morphology differences being compared.

### Assessing relationship between NeuriteNet and tracing data

In this work we also compare the relationship between the NeuriteNet scoring and neurite tracing data sets using two different approaches. First, the relationship between the Predicted Pattern Score generated by NeuriteNet and the individual tracing data metrics were assessed. Second, we employed a machine learning regression approach to investigate how well the all variables derived from the tracing data in aggregate can explain the “Predicted Pattern Score” generated by NeuriteNet for a given image using Scikit-learn [43]. The approach was similar to the classifying the neurons by their tracing data in that the same scaling and fivefold cross-validation was employed. For this approach, however, a Ridge regression model (as opposed to a Ridge classification model) was used to fit a model seeking to quantify the relationship between all metrics derived from the tracing the data to the “Predicted Pattern Score” generated by NeuriteNet for each image. An explained variance score (Scikit-learn) was calculated for each cv-group as a measure of how well the tracing data correlate with, explain the variation in, and can be used to determine the Predicted Pattern Score. The explained variance score for each of the 5 cv-groups was averaged in this assessment of both NeuriteNet and the Generic CNN.

### Concept vectors

To model the sensitivity of NeuriteNet to our tracing data metrics, we computed the Regression Concept Vectors (RCVs) and Bidirectional Relevance Scores (Br) for our models [49]. As NeuriteNet is a deep learning model operating on image inputs, the exact features encoded by the internal architecture of NeuriteNet exist in a high-dimensional space that proves inscrutable to human interpretation. The RCVs bridge this gap by modeling the relationship between these high dimensional features and our tracing data metrics in a low-dimensional space. By analyzing how changes in the RCVs relate to changes in the model predictions, we can compute the Br Scores, which show the magnitude and direction of our model's sensitivity to these concept vectors and thus the representation of our tracing data metrics in the input images. We generated Br Scores for each of the 5 cv models of NeuriteNet for the six measurements from the tracing data: Total Length, Branch Length Variation, Number of Branches, Branching Density, Alignment Index, and Alignment Variation. As with the previous methods, the tracing data were first normalized using a quantile transformation. Then the Spearman rank coefficient, ρ, was calculated to show the correlation between each measured concept (the 6 different tracing metrics) and the Predicted Pattern Score as pre-processing.

For each image with tracing data, we separately collected the output activation maps from the last convolution layer of our model. In CNN models, the activation maps are the output values coming from a single specific layer and represent the features being passed forward by that part of the model. Each activation map is generated by a single 3 × 3 kernel in the convolution layer, so each point in the map represents the strength of the presence of the feature encoded by each kernel. We applied a global maximum pooling operation to these maps to generate vectors representing the single point of strongest activation for each of the maps. We then fit a least squares linear regression model using the activation vectors from each sample as inputs and the concept measures from the same sample as target outputs. By normalizing the coefficients of the optimized linear model, we were able to find the direction of the strongest increase of the concept measures in the space of these activation vectors, giving us the RCV.

Using our computed RCVs, we then calculated Br Scores for our model, which are defined as the sensitivity of our model relative to the concept vectors of each measure [49]. To do this, we first took the partial derivative of the outputs of the model with respect to the activations of the same convolution layer that we used to find our RCVs. This partial derivative is also known as the “gradient” of the model. We used linear projection to map these gradients onto our RCV, giving us the sensitivity score representing how sensitive NeuriteNet was to the measured concepts when classifying the given sample. The magnitude of the sensitivity score gives us the rate of change of the model output relative to the concept measures, and the sign gives us the direction of the change. We repeated this process to find the sensitivity score for each sample in our dataset. Using the entire set of sensitivity scores, we computed the coefficient of variation, which is defined as the ratio of the standard deviation to the mean of the dispersion of our sensitivities. Then for each concept, the Br Score was generated by taking the ratio between the R^2^ score of the model our concept direction was drawn from and coefficient of variation of our sensitivity scores.

## Results

### Replated DRGNs grown on topographically micropatterned substrates exhibit distinct morphology

**Fig. 1:**
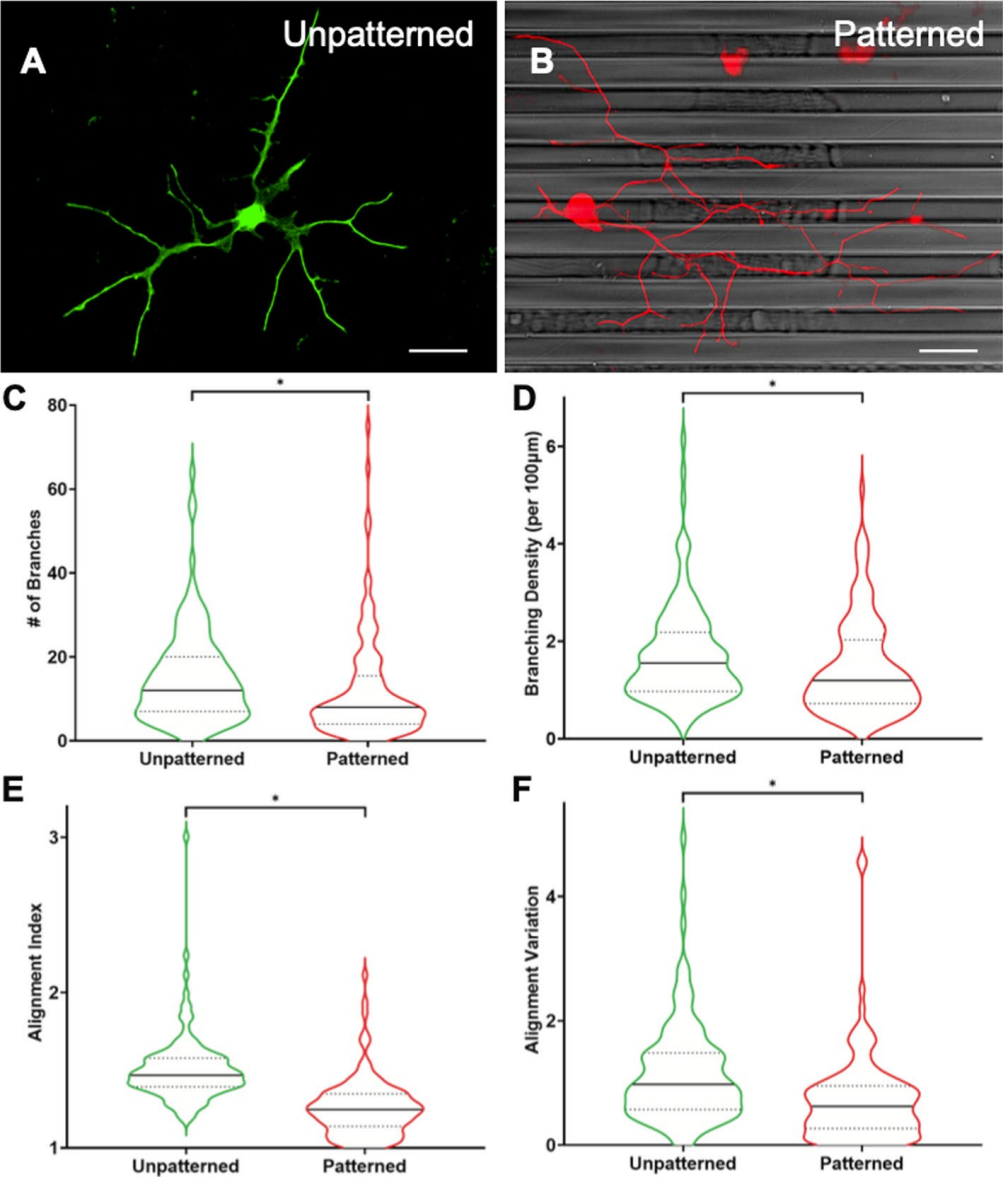
DRGNs grown on topographical micropatterns have robust differences in neurite morphology. **A**, **B** Representative images of rDRGNs grown on unpatterned (**A**) and topographically micropatterned substrates (**B**). **C** Number of Branches per DRGN is decreased in neurons grown on patterned substrates. **D** Branching Density (Neurite branches per 100 µm length) is decreased in neurons grown on patterned substrates (**E**) Alignment Index (Total neurite length divided by length in horizontal direction) shows rDRGNs align well to micropatterned substrates. **F** Alignment Variation (Variance in Alignment Index measurement of each branch from a single neuron) shows less variation in DRGNs grown on micropatterned substrates. n = 120 and 121 neurons for each comparison. Mann–Whitney test shows smaller values for the Patterned condition for each comparison (*p* < 0.01). Scale bar = 50 µm

While there are studies showing that DRGNs align to and grow in response to biophysical cues [37, 50, 51], to our knowledge, it has not been established how rDRGNs, which have a distinct growth morphology and behavior compared to “naïve” DRGNs, respond to biophysical growth cues [40, 52]. Thus, we first assessed rDRGN morphology and neurite alignment to the micropatterned substrate (Fig. 1). Neurites from rDRGNs grown on unpatterned substrates extend in random orientations (Fig. 1A), exemplified by a median Alignment Index of 1.47 (Fig. 1E), while neurites of rDRGNs grown on 4 µm amplitude topographical micropatterns align in the direction of the micropattern (Fig. 1B), illustrated by a median Alignment Index of 1.24 (Fig. 1E). This demonstrates that the rDRGNs do respond to these cues [35, 37], and furthermore the Alignment Index measurement is a robust and reproducible difference which will be useful in studying the relationship between the tracing data and the classification scoring of NeuriteNet.

We also compared 5 other measurements generated from the tracing data, where the expected findings were less clear. Interestingly, rDRGNs grown on the topographical micropatterns exhibited significant differences in 3 of the other measurements. Replated DRGNs exhibit less branching when grown on the micropatterned substrate, both in terms of Number of Branches (median of 8 ± 1.15 branches per neuron compared to 12 ± 1.03) (Fig. 1C) as well as Branching Density (median of 1.20 ± 0.09 branches per 1 compared to 1.55 ± 0.09) (Fig. 1D). Additionally, on the micropatterned substrate, rDRGNs exhibit a smaller Alignment Variation (median of 0.63 ± 0.06 compared to 0.98 ± 0.07) (Fig. 1F), meaning the individual arbors of each rDRGN on an unpatterned substrate grow in more random orientations relative to each other. We found no difference in rDRGN Total Length or Branch Length Variation between substrate groups (Additional file 2: Fig. S2).

### NeuriteNet efficaciously classifies images of neurons grown on patterned substrates from unpatterned substrates

**Fig. 2:**
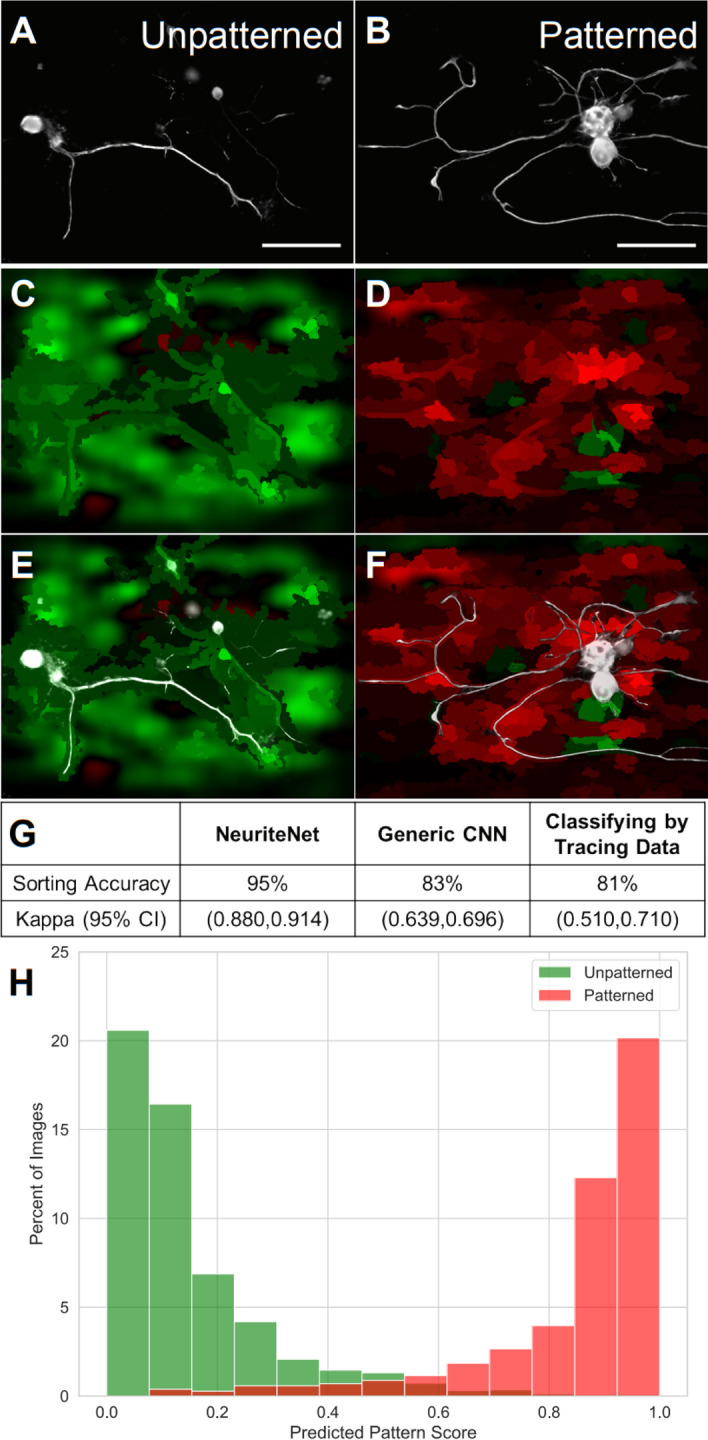
NeuriteNet effectively classifies images corresponding to DRGNs grown on unpatterned and topographically micropatterned substrates. **A**, **B** Representative images of rDRGNs grown on unpatterned (**A**) and topographically micropatterned substrates (**B**). **C**, **D** Same images as in **A**, **B** that were correctly classified as Unpatterned (**C**) or Patterned (**D**). The intensity of the color indicates the relative importance of that area. Green and red indicate areas that were used by NeuriteNet to suggest the image belonged to Unpatterned and Patterned groups, respectively. **E**, **F** The representative images of rDRGNs (**A**, **B**) with their saliency map overlayed (**C**, **D**). **G** Comparison of performance of 3 machine learning classification approaches. The percentage of total images or traces (n = 2604, 2604, 241) classified correctly as belonging to pattern and unpatterned groups is shown along with kappa statistic. **H** Fractional distribution of Predicted Pattern Scores. Color represents the actual group (pattern or unpatterned) to which the image corresponds. NeuriteNet classifies an image as “Patterned” if the Predicted Pattern Score is greater than 0.5 and to “Unpatterned” if less than 0.5. NeuriteNet classified vast majority of images correctly (the small red bar at Predicted Pattern Score of 0.4 (appears brown as it is overlaying the green) represents a miniscule fraction of patterned images falsely classified as unpatterned). Scale bar = 100 µm

Epifluorescence images of the rDRGN cultures were obtained and subjected to minimal manipulation prior to training on NeuriteNet (Fig. 2A, B). The unpatterned and patterned groups corresponded to 1421 and 1183 image of rDRGNs, respectively. NeuriteNet was trained using 80% of the images and then tested on the remaining 20%. Following repetition with fivefold cross validation, we determined that NeuriteNet classifies the images by substrate group with an accuracy of 95% and Kappa statistic with 95% confidence interval (CI) of 0.880—0.914 (Fig. 2G, H).

We compared this classification result to two other methods of screening these neurons into treatment groups. First, we used a generic CNN. This generic model uses the exact same architecture as NeuriteNet but lacks the side-branches that allow NeuriteNet to simultaneously use features from multiple levels of spatial resolution. We refer to this as the “generic” model since the remaining structure of alternative strided and non-strided convolutions is a standard architecture that has been applied to many basic classification tasks. This model classified the images of neurons with an accuracy of 83% and Kappa statistic with 95% CI of 0.639—0.696, significantly underperformed NeuriteNet (Fig. 2G). The second comparison we used was training a classification model to predict treatment group using only the neurite tracing data as input. Similarly, this approach yields accuracy better than chance (81% accuracy and Kappa statistic with 95% CI of 0.510–0.610) (Fig. 2G), but not with the same fidelity of NeuriteNet, the CNN we developed for this purpose.

After seeing its superior classification, we then assessed what drives NeuriteNet’s classification and scoring by evaluating its relation to the quantified differences in neuron morphology using multiple methods (Fig. 1). First, we generated saliency map overlays to provide insight into what regions of the image or features of the neuron NeuriteNet learned to associate with each group and qualitatively studied individual correctly classified images with Predicted Pattern Scores near to 0 or 1. The regions and features that NeuriteNet associates in each image to a particular group are color-coded (green suggests Unpatterned and red Patterned), with the intensity of the color indicating the strength of the association. Assessing these overlays suggests that NeuriteNet focuses on similar morphological differences we see quantified by the tracing data. Importantly, the approach used for these saliency maps overlay did not seek to highlight specific neuron features, but to broadly assess the relative importance of regions of the image in the classification scoring (Additional file 1: Fig. S1). For our dataset, in images of rDRGNs from the Pattern group the map highlights regions associated with horizontally aligned neurite segments in red (Fig. 2D, F). Thus, suggesting that NeuriteNet associates morphology related to Alignment Index with the Pattern group. While looking for similar tendencies in maps from neurons from the Unpatterned group, we see neurites with gradual curves or vertically aligned segments highlighted strongly green (Fig. 2C, E). These factors represent random neurite growth trajectories expected of neurons on substrates lacking any patterned growth cues.

### The tracing data are correlated with and partially explain the variation in the Predicted Pattern Score assigned to the images by NeuriteNet

**Fig. 3:**
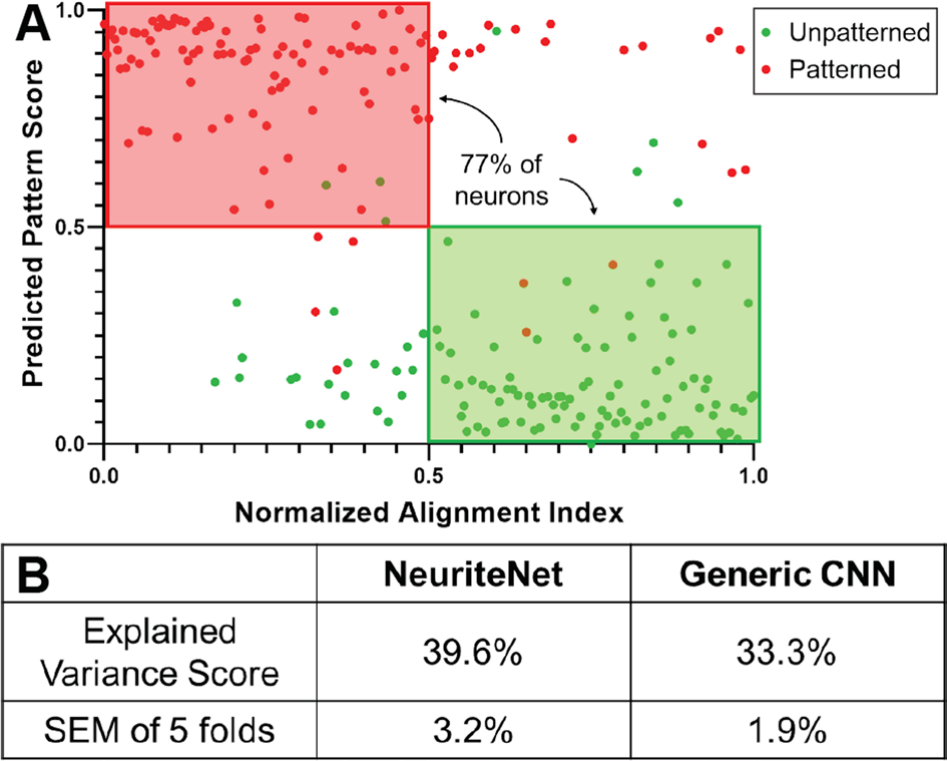
NeuriteNet scoring by pattern is partially explained by neurite tracing data and clusters with Alignment Index. **A** Scatter plot of replated DRGN plotted by their Predicted Pattern Score and rank normalized Alignment Index. Of note, for these approaches all DRGNs with tracing data were used. The scatter plot shows strong clustering between the Predicted Pattern Score and Alignment Index with Score increasing with Index decreasing. **B** Comparison of how well the two-machine learning approaches are explained by the tracing data via a ridge regression model of the tracing data seeking to explain the variance of the Predicted Pattern Score. This shows that NeuriteNet classification scoring is partially explained by the tracing data

A second approach to assess what dictates NeuriteNet’s scoring is determining if the quantified metrics correlate with or can predict the model’s scoring. We undertook two methods to evaluate this: (1) assessing the individual trends between each tracing data metric and the Predicted Pattern Score, and (2) fitting a regression model seeking to explain the variation in the model’s scoring using all of the neurite tracing metrics. First, using only test set results, we see that the Alignment Index of an individual neuron clusters with that rDRGN’s Predicted Pattern Score (Fig. 3A). Meaning that rDRGNs which align well to the pattern tend to also be classified more strongly to the Pattern condition as well as those with more random orientations of growth to Unpatterned. Though much weaker, this trend also exists for the other 3 significant differences in the neurite tracing data (Alignment Deviation, # of Branches, and Branching Density) and Predicted Pattern Score (Additional file 3: Fig. S3). Of note, the correlation analysis in Additional file 1: Fig. S3. represents the data used for the concept vector analysis. Only rDRGNs that were 1 neuron fully contained in 1 image were used to obtain these data to minimize noise and conflating factors as well as. Similarly, the score reported is an ensemble Predicted Pattern Score of all 5 cv-group models on each image so that the data used for the Br Score analysis represent a holistic and average picture of how the models interpret the rDRGNs.

A more complex approach to relate the results from the model to the neurite tracing data is utilizing fivefold cross-validation of a regression model to fit all of these tracing data measurements to the Predicted Pattern Score. In this approach, we see that when a regression model is trained to predict the Predicted Pattern Score using all measurements from the tracing data, we see that 39.6% of the variation seen in the Predicted Pattern Score can be explained using the tracing data (Fig. 3B). Thus, suggesting that all together the tracing data can explain a substantial portion, but not the majority, of the variation seen in the scoring. Furthermore, the tracing data were unable to explain as much of the variation in the Generic CNN (33.3%). Thus, suggesting that NeuriteNet’s analysis was more closely related to the tracing data.

### Br Scores demonstrate NeuriteNet classification is sensitive to tracing data

In addition to the saliency maps, which identify the relative importance of each spatial region of the image, we applied an additional method to NeuriteNet to quantify the influence of the tracing data on the classification predictions using Concept Vectors. Since the patterns of activation are inherently prone to noise, the data were constrained to minimize the noise. First, the images and tracing data utilized for this approach consisted of a random sample of neurons which were fully contained in single images. Second, to prevent features which are irrelevant from hindering the analysis, we only considered the quantified morphological measurements that had at least a weak correlation for the given classification prediction (|ρ|> 0.2) (Additional file 4: Fig. S4). This decision was made since if a concept is not correlated with a given classification prediction, then it is unlikely to represent any relevant information for that comparison.

**Fig. 4:**
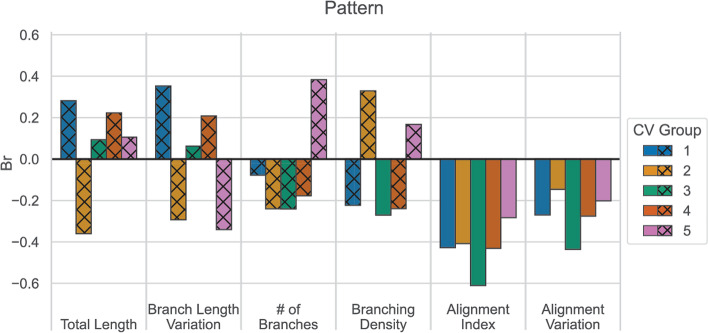
Br Score demonstrates that NeuriteNet is sensitive to changes in alignment measurements. Br Scores relating tracing data to Predicted Pattern Score calculated by NeuriteNet split by cv-group. Of note, bars with cross-hatched pattern represent data that would be removed with correlation-based thresholding approaches (data with coefficient ρ <|0.2| in Additional file 3: Fig. S3A) All cv-groups for both Alignment Index and Alignment Variation show consistent activation in the direction of patterned condition

Concept vectors to generate Br Scores were generated as a third approach to assess the relationship between NeuriteNet and the tracing data (Fig. 4). The magnitude of a Br Score increases if the model’s activations have a strong relationship with a given concept and the model shows a consistent level of sensitivity to that concept across the different images. In this, Alignment Index and Alignment Variation show consistent moderate to strong negative Br Scores for Patterned classification in all five of our validation models. Thus, indicating that there is a direct relationship between the measured Alignment Index and the features in the image learned by NeuriteNet to predict whether a neurite was grown on the Patterned substrate. Since the Br Scores are all negative, greater expression of features related to smaller Alignment Index and Alignment Variation in the input images will cause the model to be more likely to classify the image into the Patterned substrate condition. Importantly for the other four measurements, the data within the threshold described above (|ρ|< 0.2) should be excluded from interpretation and are represented by crosshatched bars on the graph (Fig. 4). Additionally, the inconsistent direction and strength of the Br Scores corresponding to the other four metrics suggest that there is no or minimal relationship between the features of an image NeuriteNet learns to associate with a condition and these length and branching data.

Br Scores were calculated in a non-biased manner for each of the 5 cross-validation models for each of the output predictions: Genotype, Sex, and Pattern (Additional file 4: Fig. S4). No clear relationship was appreciated for the Genotype or Sex analysis.

### Repeating these approaches using a separate data set further supports that NeuriteNet’s scoring is driven by/correlated with measurable, quantifiable differences in morphology

We validated the previous results by demonstrating similar findings with another dataset. For this we utilized a data set of images with neurite length measurements where neurite length is the distinguishing metric between treatment groups (Additional file 5: Fig. S5). Thus, we explored if NeuriteNet can screen images for differences in neurite length.

Before describing the data, a brief description on the biology of replating neurons and the role of Nocodazole in this assay is needed. It is theorized that there are two distinct mechanisms that are both required to allow for the regrowth of DRGN neurites after replating [40, 52]. First, is the transcription-dependent component that is encoded in the neurons during the 3 days in culture prior to replating and second is the transcription-independent, microtubule-dependent component which drives the growth in the 1 day after replating. Evidence for this is that if DRGNs are treated with 5,6-dichlorobenzimidazole riboside (DBR), a reversible inhibitor of RNA polymerase II and of transcription, in the 3 days prior to replating, growth after replating is inhibited. However, if you treat with the same drug, DBR, in the 24 h after replating there is no such inhibition of neurite growth [40]. Another finding supporting this is that if treated with Nocodazole, an inhibitor of microtubule polymerization, in the 3 days before to replating, the rDRGNs grow similar to untreated rDRGNs after replating (Before). However, since this drug blocks the microtubule-dependent component, if Nocodazole is added to the culture media for 24 h after replating, neurite growth is strongly inhibited (After) [40]. Thus, for our data set, rDRGNs treated with Nocodazole before replating have significantly longer neurites than those treated with the same drug after replating [39] (Additional file 5: Fig. S5). These two treatments will be referred to as simply Before and After in this work.

**Fig. 5:**
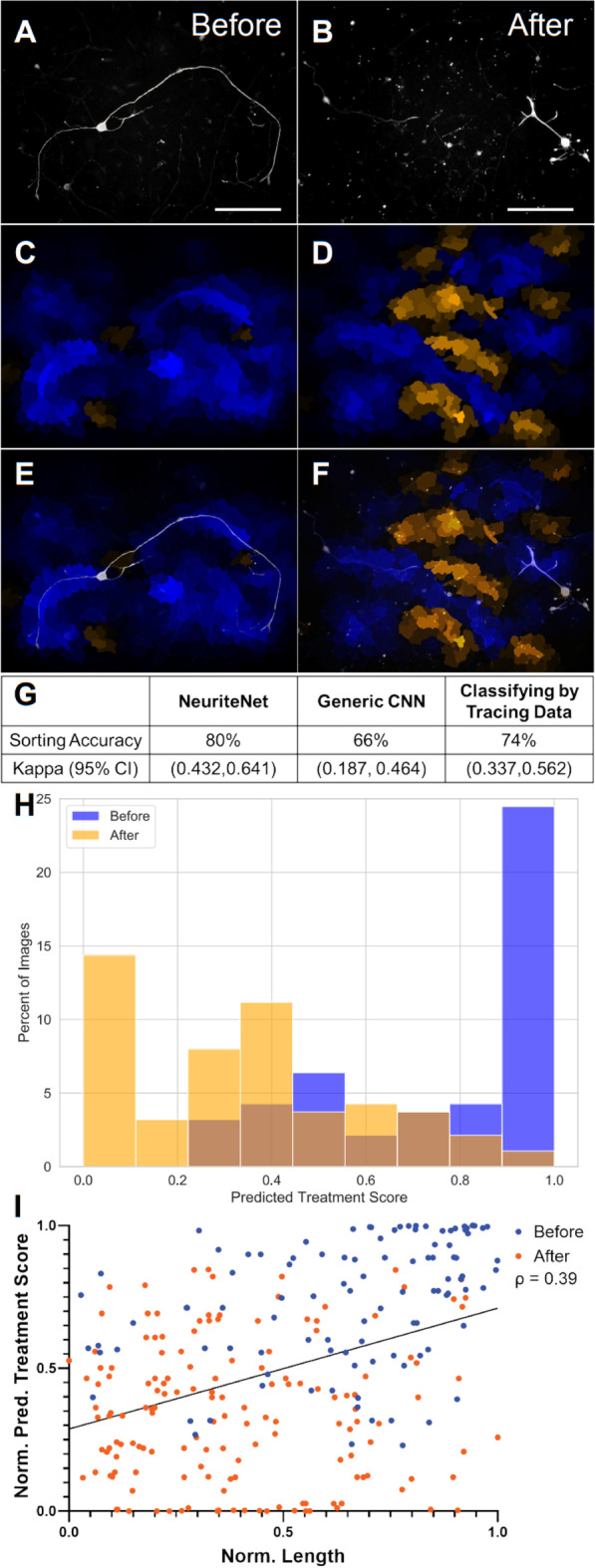
NeuriteNet effectively classifies images of DRGNs with difference in longest neurite. **A**, **B** Representative images of rDRGNs treated with Nocodazole Before (**A**) and After replating (**B**). **C**, **D** Same images as in **A**, **B** that were correctly classified as Before **C** or After **D**. The intensity of the color indicates the relative importance of that area. Blue and orange indicate areas that were used by NeuriteNet to suggest the image belonged to Before and After groups, respectively. **E**, **F** The representative images of rDRGNs (**A**, **B**) with their saliency map overlayed (**C**, **D**. **G** Comparison of performance of different classification approaches. The percentage of total images or traces (n = 178, 178, 255) classified correctly as belonging to Before or After treatment groups is shown along with kappa statistic. **H** Fractional distribution of Predicted Treatment Score. Color represents the actual group (after or before) to which the image corresponds. Of note, NeuriteNet classifies an image as “After” if the Predicted Treatment Score is less than 0.5 and to “Before” if more than 0.5. NeuriteNet classified most images correctly (the small orange bar at Predicted Treatment Score of 0.85 (appears brown as it is overlaying the blue) represents a small fraction of After images falsely classified as Before). **I** Scatter plot of rDRGN plotted by their length score and length using data normalized with a quantile transformation. Linear regression shows a modest correlation with the Predicted Treatment Score and length measurement (ρ = 0.39). Scale bar = 100 µm

The data set consists of 91 and 87 images of Before and After rDRGNs, respectively (Fig. 5A, B). Of note, this sample size is much smaller than the previous dataset (Fig. 2). The respective median rDRGN axon lengths were 663.8 µm and 378.6 µm [39] (Additional file 5: Fig. S5). Similar to the previous data set, training and testing using the three approaches was conducted using fivefold cross validation, and in this, NeuriteNet classified the images by treatment group with an accuracy of 80% (95% CI of Kappa Statistic 0.432–0.641) (Fig. 5G). As with the prior data set, this classification accuracy was compared to two other methods of screening these neurons into treatment groups. Similarly, NeuriteNet classified these images more accurately than the Generic CNN, 66% (95% CI of Kappa Statistic 0.187–0.464), and predicted treatment group using the neurite length data, 74% (95% CI of Kappa Statistic 0.337—0.562) (Fig. 5G).

Additionally, this result was validated by (1) assessing saliency map overlays of images from this comparison and (2) assessing the correlation of classification scoring with the neurite length measurements. Interestingly, the saliency maps for this classification comparison suggest a mixed picture. The map overlays for the Before treated neurons tend to highlight long elongating segments in blue (Fig. 5C, E), which is consistent with this treatment group being characterized by longer neurite length. However, the maps for the After group tend to highlight non-specific regions of the image in Orange (suggesting those regions as indicative of After). Additionally, in maps where the image is correctly classified to the After condition, the regions occupied by the neurites are somewhat overlayed in blue (Fig. 5D, F), thus even though the image as a whole is classified to After, those particular regions are more associated with Before to NeuriteNet.

While the results are mixed for these saliency maps, the After saliency maps are consistent with the classification results and the challenge presented to the model with this classification task. There are three points that aid in the interpretation of the saliency maps for this comparison. First, it is important to note that the number of images utilized is less than 10% the total of the prior comparison (178 vs. 2604). Thus, NeuriteNet had much fewer images to learn the morphological trends from. Second, if we look at the histograms of classification scoring (Fig. 5H), we can see that the model classifies a large portion of the Before images strongly (a large proportion receive a score near 1). Conversely, this is not the case with the After images as much less are scored near 0 and actually a large proportion are around 0.3 to 0.7. This indicates that the Neurite does not strongly associate a lot of the After treated neurons to either condition. Third, it is important to consider the morphological difference being studied. Here, the prominent difference in the treatment groups is the length of neurite growth. Thus, non-specific overlays with orange labeling in the background of the image may be the “correct” labeling of a well-trained model to detect shorter neurites in the After group. Taking these points into account, the saliency maps for the After condition (Fig. 5D, F) represent NeuriteNet correctly associating areas lacking elongated neurite growth to the After treatment, while regions where a neurite is present to Before. However, the seemingly non-specific regions suggestive of the After condition outweigh the regions suggestive of Before and thus the example shown was classified correctly as After.

Then in terms of comparing the scoring results to the length data, we see a modest correlation between these two values (ρ = 0.39) (Fig. 5I). This indicates a trend that Predicted Treatment Score tends to be greater in images of neurons with longer neurites. Lastly, for this data set, we were unable utilize the Br Score approach since only the longest neurite data were available to us. This one metric does not represent a sufficient variety of measures to adequately study the quantitative relationship between patterns of activation within the model and how neuron morphologycontributes to these patterns.

## Discussion

### NeuriteNet’s classification is related to, explained by, and is sensitive to changes in the tracing data

This work represents a novel pursuit to thoroughly associate the findings from CNN image analysis to neuron tracing data. In this study we demonstrate how NeuriteNet’s analysis is sensitive to and focuses on quantified morphological metrics in its analysis of neurite growth patterns. First, in an experimental system studying neurite guidance on topographically micropatterned substrates, replated DRGNs (rDRGNs) have distinct morphology relative to those on non-patterned substrates (Fig. 1). NeuriteNet detects this morphological difference, classifies the groups more accurately than other classification approaches, and reports a score between 0 and 1 (Predicted Pattern Score) related to this classification (Fig. 2G).

Multiple approaches validated NeuriteNet’s findings, including: (1) saliency map overlays highlighting regions associated with horizontally aligned neurite segments in the pattern group and gradually curved or vertically aligned segments in the unpatterned group (Fig. 2C, D), (2) Alignment Index and Predicted Pattern Score clustering (Fig. 3A), (3) the tracing data explaining a substantial portion the classification results (Fig. 3B), and (4) NeuriteNet classification being sensitive to changes in the Alignment Index measurement (Fig. 4). In combination, these data imply that in its image classification analysis, NeuriteNet learns features in the images related to quantifiable components of neuron morphology that are captured in the tracing data, such as Alignment Index.

To study these findings further, we applied NeuriteNet to another dataset where we corroborated the model’s sensitivity for this application. The second dataset studied the effect of treatment with nocodazole, an inhibitor of microtubule polymerization, on rDRGN growth at selective times during replating to either have no effect on (Before replating) or to inhibit (After replating) neurite growth (Additional file 5: Fig. S5). As with the dataset assessing neurite morphology on micropatterned substrates, NeuriteNet correctly assigns images to Before or After treatment group with accuracy greater than other comparable classification approaches (Fig. 5G). The follow up approaches substantiate these classification results with (1) saliency map overlays highlighting long elongating segments in the Before treated neurons (Fig. 5C) and (2) the length measurements and Predicted Treatment Score (the score related to NeuriteNet’s classification) demonstrating correlation (Fig. 5I). While the specificity of the maps and the degree of correlation are not as robust as the previous analysis of rDRGNs being classified by substrate, this was an expected finding due to the experimental setup. First, the dataset used for this had fewer images and only one measurement associated with the image (only 178 images compared to 2604 in the previous study). This limited the ability of NeuriteNet to learn the characteristic features of the neurons from each treatment group and effected the degree of correlation we observed. Second, the saliency maps of the After group showing non-specific overlays is also an anticipated finding. In addition to the specificity being limited by the small sample size, the morphology being compared also complicates this saliency map approach. If the only difference in the treatment groups is the length of the neurite, non-specific overlays in the background of the images, where there are not neurites, would be the expected pattern seen in the After group with shorter neurites. Overall, these data imply that NeuriteNet is also able to distinguish differences in neurite length due to drug treatment in its classification.

Taken together, these data demonstrate that in their analysis CNNs (1) assess quantifiable morphological differences in neurite growth, (2) consider distinguishing factors that may not be encompassed by traditional neuron morphology analyses, and (3) function as flexible and efficient neurite analysis tools. Additionally, this work serves as an outline for those studying neuron morphology in terms of how to integrate CNNs in their study of neurite growth. For example, this work highlights various methods for how to validate the application of CNN models for assessing neurite growth by using saliency map overlays, comparing the scoring behind the classification to tracing data, and Concept Vectors (Br Scores). Additionally, this work suggests the CNN architecture best suited for similar tasks is one with side branches as NeuriteNet appears effectively suited for studying neuron morphology.

### NeuriteNet detects differences in neuron morphology not encompassed by the tracing data

Our quantitative methods to validate NeuriteNet suggest that our neurite tracing data only explain a portion of the variation of the model’s classification scoring results. Specifically, about half of what is driving the classification decision is unexplained by the tracing data in the Patterned versus Unpatterned comparison (Fig. 3, Additional file 3: Fig. S3).

The saliency map overlays imply that the model is focusing on regions associated with neuron morphology in making this classification (Fig. 2C, D). In particular, while there is some non-specific labeling in the saliency maps, the non-specificity is not consistent within each treatment group. Thus, as a whole this assessment suggests that regions of images unrelated to neuron morphology, which hypothetically tend to correlate between groups, are only a minor factor in NeuriteNet’s classification since there is not a clear trend in the background of the saliency map. Consequently, to explain the remainder of the variation in the model’s scoring, NeuriteNet must be focusing on aspects of neuron morphology, that predictably vary in the treatment groups, which are not encompassed by our specific neurite tracing data. There are many morphological metrics that our tracing data does not encompass, including, but not limited to (1) the relation of the arbors spatially (i.e., to the soma or other neurites), (2) neurite thickness, or (3) micro-scale curvature of individual neurites. All together these metrics may predictably correlate in the treatment groups here and if so, they would explain more of the variation in the model and how NeuriteNet can outperform the tracing data.

Overall, this indicates that when generating its “score”, NeuriteNet thoroughly assesses neuron morphology by considering metrics easily evaluated by neurite tracing as well as also considers features that may be difficult to quantify and or not traditionally assessed. This conclusion motivates similar work to understand what drives CNN models (i.e., can neurite thickness differences be reliably detected by a model like NeuriteNet). Additionally, based on our assessment showing neurite tracing data only partially explains the difference between the Patterned and Unpatterned treatment groups, this should encourage researchers to incorporate more thorough and broader analyses when studying the morphology of neurite growth.

### Replated DRGNs are guided by and branch less on the biophysical micropattern

The results also demonstrate novel biological findings of the effect of micropatterned biophysical substrates on neuron morphology. Neurites from rDRGNs are guided by biophysical cues and seem to strongly align to the micropatterns consisting of parallel ridges and grooves (Fig. 1E). Thus, this implies that the elongating growth phenotype displayed by rDRGNs does not override their innate ability to sense and be guided by biophysical micropatterned growth cues. Additionally, the results show that rDRGNs branch less when grown on these repeating rows of ridges and grooves (Fig. 1C, D). Other work has explored using biophysical cues to control neurite branching with analogous findings of branching decreasing on similarly patterned substrates [53, 54]. These data imply that the micropatterns not only direct neurite growth; they also limit branching events. The cellular mechanisms that underlie this reduction in branching remain unknown. Thus, both NeuriteNet and biophysical growth cues represent tools to study neurite guidance and branching decisions.

### Future work using NeuriteNet to study neurite guidance, morphology, and branching

CNNs provide powerful tools for high throughput image analyses as they are much faster than other holistic approaches (Sholl [55], neuron tracing [31]), while also being sensitive to various morphological differences. In this work, NeuriteNet distinguishes and scores neuron morphology by treatment groups more accurately than a “Generic CNN” (Additional file 6: Fig. S6). Since the generic CNN has the same architecture of NeuriteNet but lacks the side-branches, this suggests that the side-branches in NeuriteNet enable a more accurate assessment of neuron morphology. Side branching has been shown to be key for optimizing CNNs for other image analyses which require multi-scale assessment [56–58]. These previous studies motivated the incorporation of side-branches into NeuriteNet as well as support the idea that these branches enable NeuriteNet to analyze features from multiple levels of spatial resolution of neuron morphology. Based on the improvements in model accuracy with the incorporation of the side-branches, the field would benefit from more research into which components of CNN architecture (such as side-branch structure or number) enable a model to effectively analyze neuron morphology.

An additional aim for those using CNN image analysis models to study images with quantified measurements could be to better investigate how saliency maps relate to the tracing data in a quantified manner. This endeavor would allow for better feedback on the model specificity as well as could enable improved saliency map overlays. In the context of studying neuron morphology, one could simply assess the specificity of the overlayed map’s signal to the location of neuron tracing on an image to determine the fidelity of the model to only considering the neuron morphology. Additionally, more advanced approaches could compare the saliency maps to the specific feature of neuron morphology (branch, turn, aligned segment) present at that region of the image. This work would provide a platform to further optimize CNN function and saliency map development for the study of the morphological parameters of neurite growth.

In addition to more research into how the model functions, we could also pursue expanding the application and outputs of models like NeuriteNet. Avenues of this future work include assessing the applicability of NeuriteNet to study other neurons with complex morphology or innervation patterns of tissue sections. We expect that NeuriteNet is ideally suited to analyze neurons with morphologies more complex than DRGNs, such as hippocampal or Purkinje neurons [59, 60], or neurite growth in vivo*,* such as innervation in the dermis or spiral ganglion neuron regeneration in the cochlea [61–63].

Lastly, the ideal output of NeuriteNet would be a quantifiable metric(s) to describe neuron morphology akin to what many in this field are accustomed to and prefer, as opposed to the score reported by NeuriteNet here (Figs. 2H, 5H, Additional file 6: Fig. S6). Therefore, an additional goal is to adapt NeuriteNet to directly predict the desired morphological components in its classification. Others in the field have explored similar tasks in other applications of CNN image analyses [20, 64, 65].

## Conclusion

In effectively classifying and scoring morphological differences in heterogeneous group of neurons, NeuriteNet focuses on quantifiable traits of the neuron that represent the differences distinguishing those groups. In its analysis, NeuriteNet demonstrates greater classification accuracy than neurite tracing data or a CNN lacking side-branching. Through this work, NeuriteNet also confirms a novel finding that rDRGNs exhibit a distinct, aligned morphology with decreased branching when grown on and guided by biophysical cues. Additionally, while NeuriteNet’s scoring and patterns of activation are sensitive to the quantified neuron tracing data, NeuriteNet also appears to consider morphological features not encompassed by our neuron tracing data. Though there are some features not explained by the tracing data that NeuriteNet assesses in its analysis, importantly our work indicates that NeuriteNet is focuses on and is activated by relevant measurements of neuron morphology to generate quantitative scoring that is related to neurite growth patterns.

### Supplementary Information

**Additional file 1: Fig S1. Saliency maps are generated using a combination of iterative occlusion and graph-based image segmentation**. Input images are first processed using a Sobel filter. Following this, a rough saliency map is generated using iterative occlusion. Independently of the occlusion, the image is divided into component pieces using graph-based Felzenswalb segmentation. The rough saliency map and the segmented image are combined using XRAI to generate a saliency map with sensitivity to finer details in the input image.

**Additional File 2: Fig S2. DRGNs grown on topographical micropatterns have no difference in total length or branch length variation**. (A) rDRGNs have no difference in total neurite length when grown on patterned or unpatterned substrates. (B) rDRGNs have no difference in the variation in branch lengths when grown on patterned or unpatterned substrates.

**Additional File 3: Fig S3. Correlation plots of tracing measurements and Pattern Score**. Linear regressions comparing tracing data and Pattern Scoring using data normalized with a quantile transformation. A. Total Length shows no correlation with Predicted Pattern Score (ρ = 0.05). B. Branch Length Variation shows no correlation with Predicted Pattern Score (ρ = 0.05). C. Number of Branches shows small correlation with Predicted Pattern Score (ρ = 0.14). D. Branching Density shows small correlation with Predicted Pattern Score (ρ = 0.14). E. Alignment Index shows strong correlation with Predicted Pattern Score (ρ = 0.53). F. Alignment Variation shows modest correlation with Predicted Pattern Score (ρ = 0.30).

** Additional File 4: Fig S4. Assessment of NeuriteNet sorting by all three binary assessments**. A. Spearman rank coefficient (ρ) comparing the tracing data with the sorting scores for all 3 comparisons of interest by cv-group. The threshold lines of ρ > |0.2| represents data that was filtered prior to Br Score calculation to minimize noise. B. Br Scores relating tracing data to sorting scores calculated by NeuriteNet split by cv-group. Data with cross-hatched pattern represent data that would be removed with thresholding approaches (data with coefficient ρ < |0.2| in Fig S3A.)

** Additional File 5: Fig S5. rDRGNs treated with Nocodazole Before replating have longer axons than rDRGNs treated After replating**. (A) Schematic showing replating process and that DRGNs treated with nocodazole After replating have decreased neurite growth comparing to DRGNs treated with nocodazole Before replating. (B)Longest axon tracing data shows longer axons in Before than After (n = 103 & 157). Mann-Whitney test p  < 0.001.

** Additional File 6: Fig S6. Comparison of the scoring distribution of NeuriteNet with the Generic CNN for both sorting tasks**. (A) NeuriteNet sorts rDRGNs on patterned and unpatterned substrates at an accuracy of 95%. (D) NeuriteNet sorts rDRGNs treated with Nocodazole Before replating and After replating at an accuracy of 80%. (C) Generic CNN sorts rDRGNs on patterned and unpatterned substrates at an accuracy of 83%. (D) Generic CNN sorts rDRGNs treated with Nocodazole Before replating and After replating at an accuracy of 66%.

## Data Availability

All data and materials relevant to the publication are freely available online. Methods are available through GitHub: https://github.com/Mullans/NeuriteNet. Data from this work is on Zenodo: https://doi.org/10.5281/zenodo.7314256.
