# Supplementary figures and images for: Sensitivity of CNN image analysis to multifaceted measurements of neurite growth
Source: BMC Bioinformatics. 2023 Aug 24;24:320. doi: 10.1186/s12859-023-05444-4 (PMC10464248; doi:10.1186/s12859-023-05444-4)

Source Image

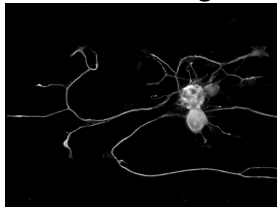

Sobel

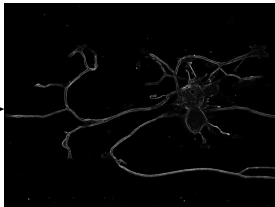

XRAI

Occlusion

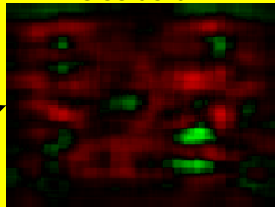

Felzenswalb

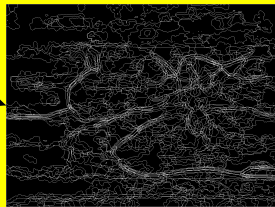

Merge

Saliency Map

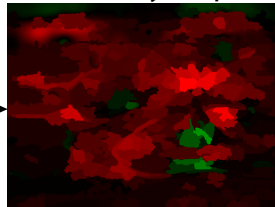

Supplement: Supplementary file 1 — Additional file 1: Fig S1. Saliency maps are generated using a combination of iterative occlusion and graph-based image segmentation. Input images are first processed using a Sobel filter. Following this, a rough saliency map is generated using iterative occlusion. Independently of the occlusion, the image is divided into component pieces using graph-based Felzenswalb segmentation. The rough saliency map and the segmented image are combined using XRAI to generate a saliency map with sensitivity to finer details in the input image. [file 12859_2023_5444_MOESM1_ESM.pdf]

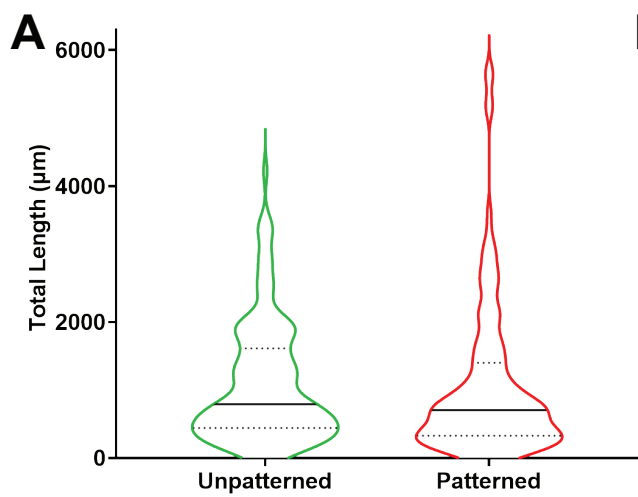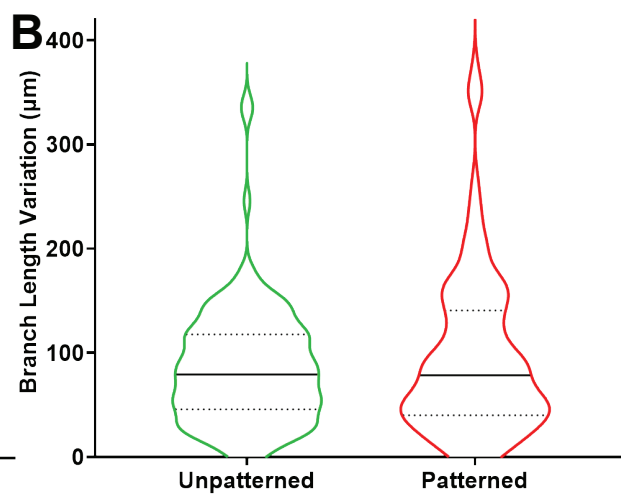

Supplement: Supplementary file 2 — Additional File 2: Fig S2. DRGNs grown on topographical micropatterns have no difference in total length or branch length variation. (A) rDRGNs have no difference in total neurite length when grown on patterned or unpatterned substrates. (B) rDRGNs have no difference in the variation in branch lengths when grown on patterned or unpatterned substrates. [file 12859_2023_5444_MOESM2_ESM.pdf]

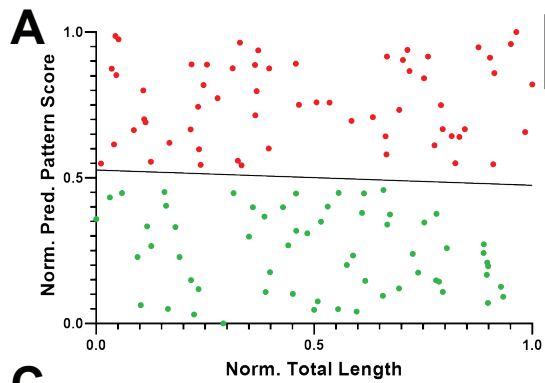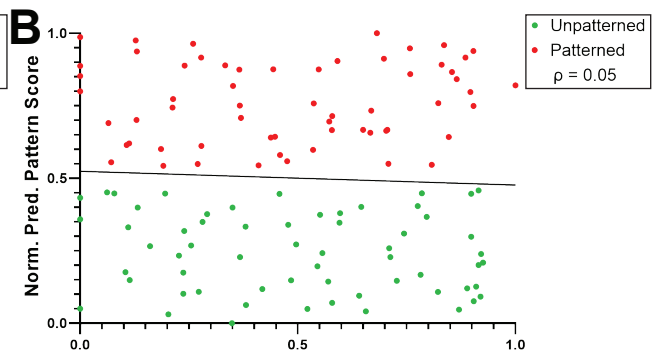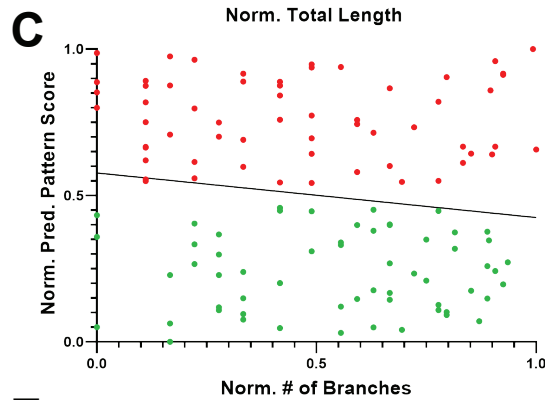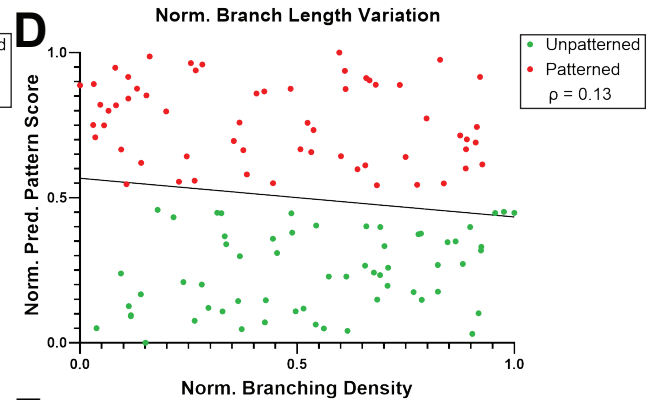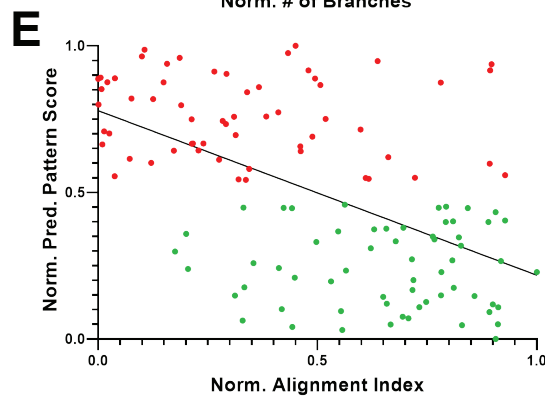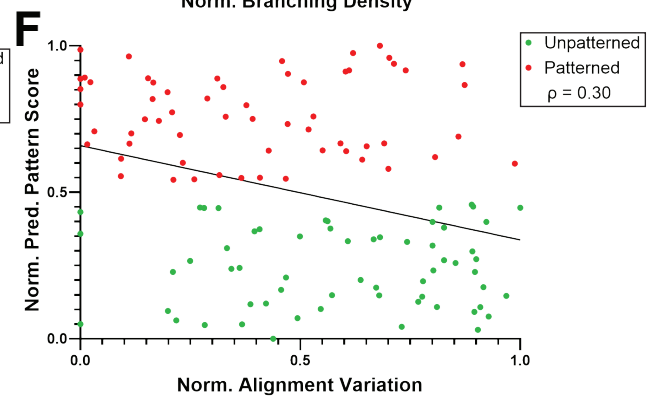

Supplement: Supplementary file 3 — Additional File 3: Fig S3. Correlation plots of tracing measurements and Pattern Score. Linear regressions comparing tracing data and Pattern Scoring using data normalized with a quantile transformation. A. Total Length shows no correlation with Predicted Pattern Score (ρ = 0.05). B. Branch Length Variation shows no correlation with Predicted Pattern Score (ρ = 0.05). C. Number of Branches shows small correlation with Predicted Pattern Score (ρ = 0.14). D. Branching Density shows small correlation with Predicted Pattern Score (ρ = 0.14). E. Alignment Index shows strong correlation with Predicted Pattern Score (ρ = 0.53). F. Alignment Variation shows modest correlation with Predicted Pattern Score (ρ = 0.30). [file 12859_2023_5444_MOESM3_ESM.pdf]

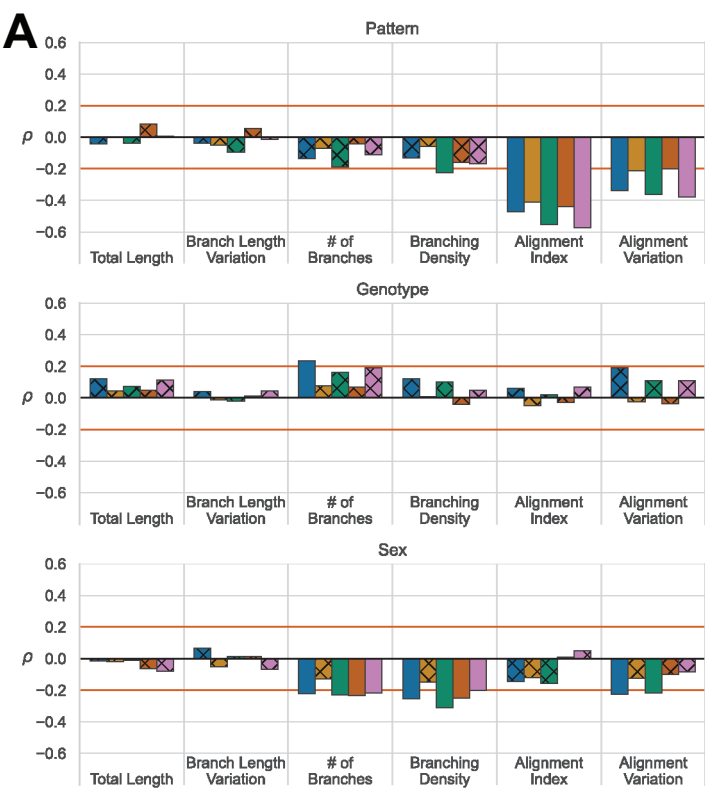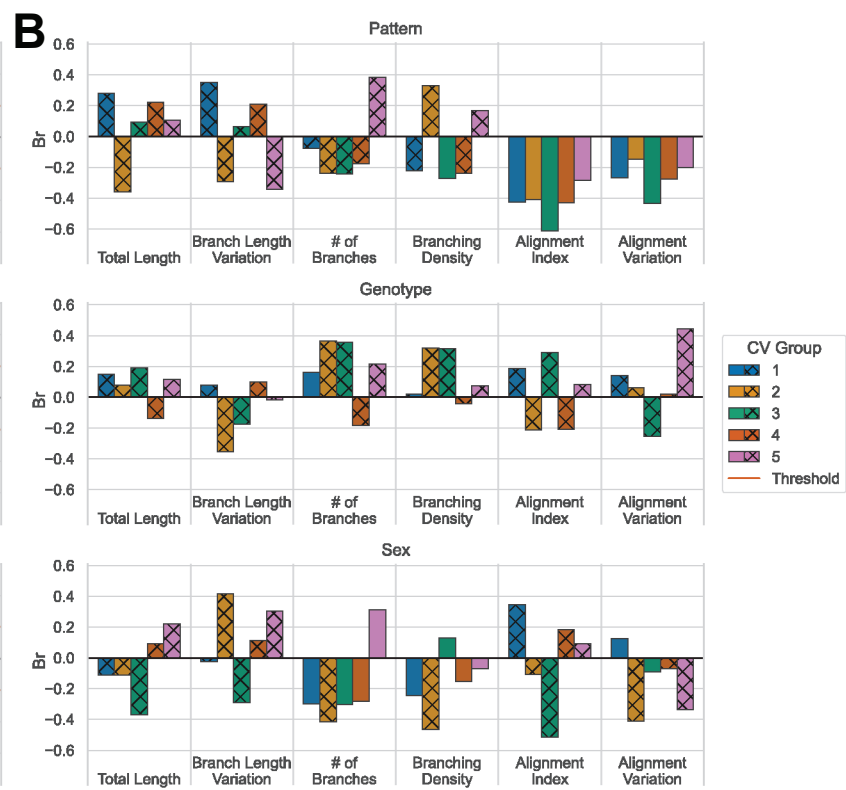

Supplement: Supplementary file 4 — Additional File 4: Fig S4. Assessment of NeuriteNet sorting by all three binary assessments. A. Spearman rank coefficient (ρ) comparing the tracing data with the sorting scores for all 3 comparisons of interest by cv-group. The threshold lines of ρ > |0.2| represents data that was filtered prior to Br Score calculation to minimize noise. B. Br Scores relating tracing data to sorting scores calculated by NeuriteNet split by cv-group. Data with cross-hatched pattern represent data that would be removed with thresholding approaches (data with coefficient ρ < |0.2| in Fig S3A.) [file 12859_2023_5444_MOESM4_ESM.pdf]

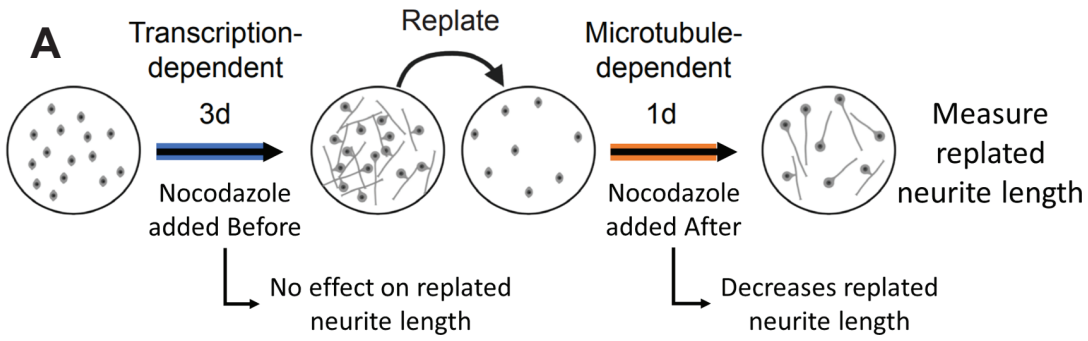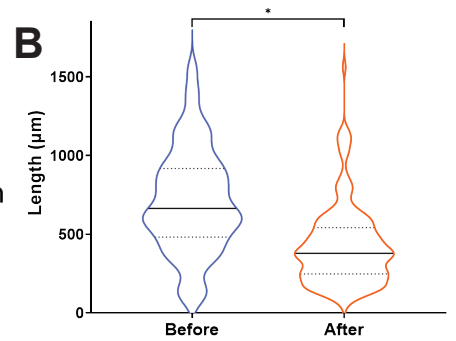

Supplement: Supplementary file 5 — Additional File 5: Fig S5. rDRGNs treated with Nocodazole Before replating have longer axons than rDRGNs treated After replating. (A) Schematic showing replating process and that DRGNs treated with nocodazole After replating have decreased neurite growth comparing to DRGNs treated with nocodazole Before replating. (B)Longest axon tracing data shows longer axons in Before than After (n = 103 & 157). Mann-Whitney test p < 0.001. [file 12859_2023_5444_MOESM5_ESM.pdf]

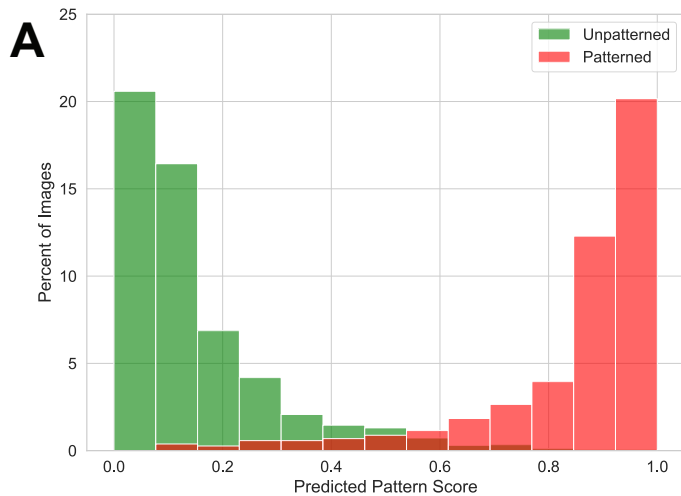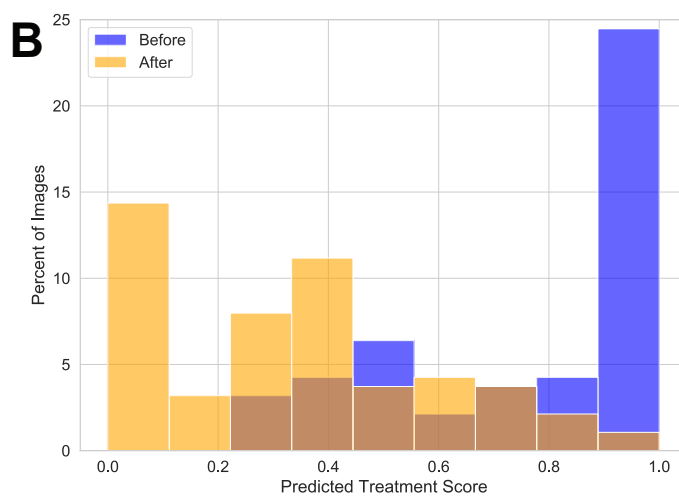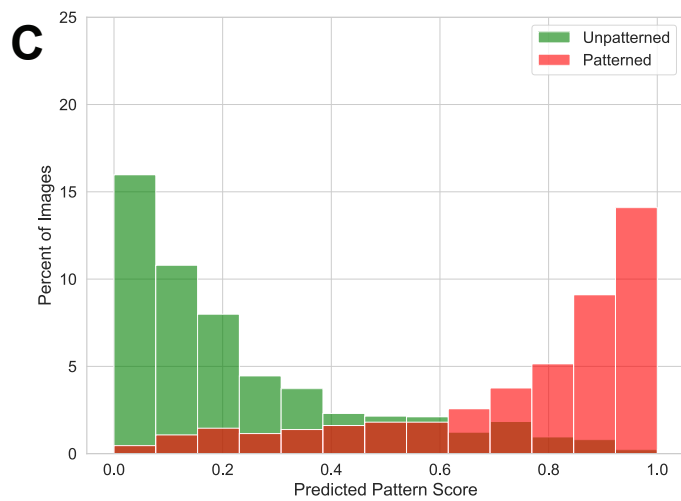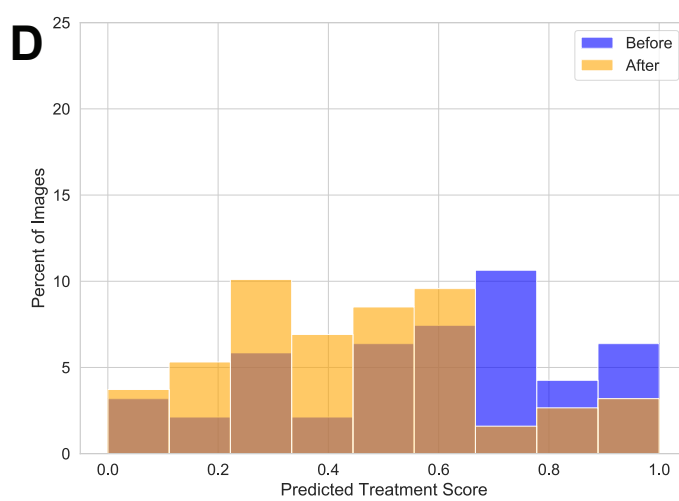

Supplement: Supplementary file 6 — Additional File 6: Fig S6. Comparison of the scoring distribution of NeuriteNet with the Generic CNN for both sorting tasks. (A) NeuriteNet sorts rDRGNs on patterned and unpatterned substrates at an accuracy of 95%. (D) NeuriteNet sorts rDRGNs treated with Nocodazole Before replating and After replating at an accuracy of 80%. (C) Generic CNN sorts rDRGNs on patterned and unpatterned substrates at an accuracy of 83%. (D) Generic CNN sorts rDRGNs treated with Nocodazole Before replating and After replating at an accuracy of 66%. [file 12859_2023_5444_MOESM6_ESM.pdf]
